# Optimizing Model Performance and Interpretability: Application to Biological Data Classification

**DOI:** 10.3390/genes16030297

**Published:** 2025-02-28

**Authors:** Zhenyu Huang, Xuechen Mu, Yangkun Cao, Qiufen Chen, Siyu Qiao, Bocheng Shi, Gangyi Xiao, Yan Wang, Ying Xu

**Affiliations:** 1 College of Computer Science and Technology, Jilin University, Changchun 130012, China; zhenyuh19@mails.jlu.edu.cn (Z.H.); xiaogy19@mails.jlu.edu.cn (G.X.); 2 Systems Biology Lab for Metabolic Reprogramming, Department of Human Genetics and Cell Biology, School of Medicine, Southern University of Science and Technology, Shenzhen 518055, China; m250921296@gmail.com (X.M.); chenqf829@foxmail.com (Q.C.); shibc22@mails.jlu.edu.cn (B.S.); 3 School of Mathematics, Jilin University, Changchun 130012, China; 4 School of Artificial Intelligence, Jilin University, Changchun 130012, China; caoyk20@mails.jlu.edu.cn

**Keywords:** feature gene selection, model selection, machine learning, interpretability

## Abstract

This study introduces a novel framework that simultaneously addresses the challenges of performance accuracy and result interpretability in transcriptomic-data-based classification. **Background/objectives**: In biological data classification, it is challenging to achieve both high performance accuracy and interpretability at the same time. This study presents a framework to address both challenges in transcriptomic-data-based classification. The goal is to select features, models, and a meta-voting classifier that optimizes both classification performance and interpretability. **Methods**: The framework consists of a four-step feature selection process: (1) the identification of metabolic pathways whose enzyme-gene expressions discriminate samples with different labels, aiding interpretability; (2) the selection of pathways whose expression variance is largely captured by the first principal component of the gene expression matrix; (3) the selection of minimal sets of genes, whose collective discerning power covers 95% of the pathway-based discerning power; and (4) the introduction of adversarial samples to identify and filter genes sensitive to such samples. Additionally, adversarial samples are used to select the optimal classification model, and a meta-voting classifier is constructed based on the optimized model results. **Results**: The framework applied to two cancer classification problems showed that in the binary classification, the prediction performance was comparable to the full-gene model, with F1-score differences of between −5% and 5%. In the ternary classification, the performance was significantly better, with F1-score differences ranging from −2% to 12%, while also maintaining excellent interpretability of the selected feature genes. **Conclusions**: This framework effectively integrates feature selection, adversarial sample handling, and model optimization, offering a valuable tool for a wide range of biological data classification problems. Its ability to balance performance accuracy and high interpretability makes it highly applicable in the field of computational biology.

## 1. Introduction

Machine learning encompasses a variety of techniques aimed at identifying a mapping from a feature space to labeled outputs while optimizing a given objective function. These methods have found wide application in data analysis across a range of domains that generate large volumes of data. Various machine-learning algorithms, such as neural network-based methods [1], support vector machine (SVM) [2], regression models [3], and decision tree-based methods [4,5], are commonly employed for addressing different data-analysis challenges. It is widely recognized that the effectiveness of these algorithms can vary depending on the problem at hand, as highlighted by the “No Free Lunch Theorem” [6]. Hence, a common issue faced by data-analysis practitioners, particularly those who are not familiar with the detailed mathematics underlying the relevant algorithms, is: which of these algorithms makes the best choice for his/her problem?

Effective algorithm selection must address three key issues: (1) interpretability—whether the model provides biologically meaningful insights into the features [7]; (2) predictive performance—whether the model achieves robust accuracy [8]; and (3) data suitability—whether the model aligns well with the characteristics of the data [9]. Current approaches to feature interpretability often rely on feature importance metrics [10,11], which tend to overlook interactions among features. In biological systems, synergistic interactions can be more informative than the contribution of individual features [12,13]. Similarly, while performance metrics such as the F1 score and AUC are useful and serve as good indicators for model selection, they may not adequately capture the true predictive capability for omics data, where high label noise is common [14].

To address this challenge, we propose a framework that integrates interpretable feature selection and model selection, specifically designed for omics data analyses. Our approach is grounded in the principle that feature selection should be based on target-related pathways, which are not only strongly discriminative but also enable meaningful interpretation of the features selected [15], as high interpretability is crucial for understanding the biological significance of the models built. Additionally, the framework incorporates adversarial samples [16,17] to assess the sensitivity of both the features and models, effectively addressing the inherent uncertainties that omics data may have [18,19].

Overall, our framework covers feature selection and model selection with the goal of identifying a model that can differentiate samples sharing common yet hidden labels, based on a concise set of interpretable features. The framework consists of two main components: one for selecting interpretable features and another for evaluating models that best align with the data. To achieve the framework’s objectives, we ultimately develop a stacking meta-classifier using the outcomes of model selection. In this work, we explore five machine learning models, with the flexibility to incorporate additional models in future applications.

This study focuses on analyzing transcriptomic data in the context of human disease research, specifically utilizing cancer tissue transcriptomic data from the TCGA database [20]. We illustrate the effectiveness of our framework by applying it to various cancer data analysis problems. For simplicity, throughout the paper, we use the terms machine-learning algorithms and models interchangeably. In conclusion, the key contributions of our work are as follows:

1. We propose a novel framework that integrates interpretable feature selection and robust model selection by incorporating adversarial samples, thereby enhancing both predictive performance and biological interpretability.
2. We introduce a domain-specific feature selection strategy based on target-related pathways in transcriptomic data, which outperforms conventional general-purpose methods.
3. We develop a stacking meta-classifier that demonstrates superior performance in both binary and ternary classification problems, underscoring its potential for broad applications in omics data analysis.

## 2. Materials and Methods

### 2.1. Datasets

This study presents two classification tasks to showcase the effectiveness of the proposed method. The first task involves identifying genes whose expression levels can effectively differentiate between 368 melanoma samples that have metastasized and 102 samples that have not, with data sourced from the TCGA [21]. Due to the occurrence of distant non-lymph node metastasis, some of the primary cancer samples in this dataset may actually be metastasized, but they have been labeled as primary cancer, making them adversarial samples [22]. The second task focuses on distinguishing genes whose expression levels can separate 436 primary cancer samples based on their respective metastatic sites: 207 to the liver, 177 to the lung, and 52 to the bone, with all data also from TCGA. For these sites, the metastatic cancer cells might remain dormant, introducing some uncertainty in the accuracy of metastatic site identification [23]. For clarity, we refer to these two problems as the binary and ternary classification tasks, respectively, in the Section 3.

Each dataset includes expression data for 60,499 genes, as provided by GENCODE v23 [24], while enzyme-related genes are sourced from the HumanCyc database, totaling 2453 genes [25]. All gene expression data are standardized to TPMs (transcripts per million) for consistency.

### 2.2. Basic Machine Learning Models

This study evaluates the predictive performance using five existing classification models: logistic regression (LR) [3], LightGBM (LGBM) [5], random forest (RF) [26], support vector machine (SVM) [27], and XGBoost (XGB) [4], which is readily extendable to include more classification models. To ensure classification generality, the dataset for each classification problem is split with a random seed, currently using 42, and all models undergo 5-fold cross-validation (CV).

### 2.3. Feature Selection for Transcriptomic-Data-Based Classification

We begin the feature selection process by focusing on the 2453 enzyme genes [25]. The objective is to identify a small subset of genes whose expression profiles possess sufficient discriminative ability to group the labeled samples into categories, each corresponding to a specific label, while ensuring that the biological roles of these genes provide meaningful insight into the separation.

**Assessing a gene (feature)’s importance to a classification problem**: Consider a set of samples **S** = {$s_{1}$, …, $s_{n}$}, each having a label from $L=\{l_{1}$, …, $l_{m}$}, denoted as ***L(S)***, with each $s_{i}$ representing the expressions of K genes. A classification problem is to find a subset of k genes G = ${\{g}_{1}$, …, $g_{k}\}$ so that a logistic regression of G’s expressions can accurately predict ***L(S)***. The level of contribution by gene $g_{i}$ to the performance by the regression model is measured using the standard *importance score* [28], defined as the absolute value of the coefficient of the $g_{i}$ term in the model. Using this approach, one can assess the importance level of each gene in G to the classification problem and remove those with no or little importance when solving the classification problem.

While the approach has been used widely, its weakness has been pointed out and alleviated by multiple authors using different techniques [29,30]; where the issue lies in that it tends to underestimate the importance of informative genes whose expressions weakly and nonlinearly correlate with ***L(S)*** [31]. A general strategy for overcoming the weakness is through applying a technique called *non-importance scoring* [32], which is based on the following premise: The importance value of a gene having true discerning power to the classification problem as defined above, called **C**, generally goes down in a modified classification problem with mislabeled samples, denoted as **C’**, which is the same as **C** except that the labels of a subset **S**’ of **S** are permuted. An execution of this strategy can be conducted through comparing the importance value of the gene in **C** vs. the importance-score distribution across a set **{C’}**, each having a different permutation of the labels of **S**’, and the gene is considered as *important* if its importance score to C is generally higher than those with partially permutated labels.

**Differential gene filtering:** DESeq2 [33] was used to identify, among genes deemed to be important, differentially expressed genes between samples with one label and those with others. Genes with a fold change (|FC|) ≥ 1.5 and an adjusted *p*-value (adj. *p*-value) < 0.05 were considered differentially expressed. Then, a pathway enrichment analysis was conducted using ClusterProfiler software v3.20 [34] with differentially expressed genes. Only pathways with an adjusted *p*-value < 0.05 for the enrichment were selected for further analyses.

**Representative pathway identification:** For a given enriched pathway, let *X* represent the gene expression matrix, where each column corresponds to a gene and each row represents a sample. The covariance matrix of *X*, denoted as C, is calculated as:

(1) $$C=\frac{1}{n-1}(X-\bar{X}{)}^{T}\cdot (X-\bar{X})$$

where *n* is the total number of samples, and X is the vector of mean expressions for each gene across all samples in *X*. Significantly enriched pathways are required to contain no fewer than five genes.

Let ${PC}_{1}$ be the first principal component of *X*, which captures the maximum variance in the pathway’s gene expression data. Let V be the variance of ${PC}_{1}$. In our pathway-based feature selection, we select pathways first, whose V values are sufficiently large. However, the V value is pathway size-dependent, which tends to go down with the increase in the number of genes of a pathway. Hence a V value-based selection procedure requires normalization with respect to the pathway size. Based on our preliminary analyses, we first consider only pathways with V > 0.7. To derive an empirical relationship between V values and pathway sizes, we have considered the density distribution of pathway size *k* across these pathways, revealing that it follows a power law distribution [35] α=0.5. Let $k_{m}$ be the median of this distribution. For a pathway with size *k* and variance V, we normalize the V value as follows:

(2) $$V’=V\times {\left(\frac{k_{m}}{k}\right)}^{\alpha }.$$

**Selection of representative genes for a selected pathway:** For each gene $g_{i}$ within the pathway, denote $v_{i}$ as the loading of gene *i* on the first principal component (PC1), and $\left|v_{i}\right|$ represents the contribution of *i* to PC1. The total contribution to PC1 is given by the sum $\sum_{i=1}^{t}|v_{i}|$, where *t* is the total number of genes in the pathway. For simplicity, we assume that the values of $v_{1}$, $v_{2}$, …, $v_{t}$ are sorted in descending order based on the magnitude of $\left|v_{i}\right|$. Let *s* be the smallest index such that:

(3) $$\sum_{i=1}^{s}|v_{i}|\;>\;95\%\;\sum_{i=1}^{t}|v_{i}|.$$

We leave out genes $v_{s+1}$ ... $v_{t}$ from further consideration. To address redundancy in the enriched pathways, we remove parent pathways when parent–child relationships are detected, retaining only the more specific child pathways.

**Filtering of genes insensitive to adversarial samples:** Similarly, a gene-sensitivity testing method was employed to assess the robustness of the selected genes against *adversarial* samples, defined as samples with their labels randomly permutated. Across 100 random permutations, we obtained the probability density distribution of each gene’s importance score. Genes that showed important scores lower than those under true labels in at least 95 of these 100 iterations were retained for final analysis, ensuring insensitivity to adversarial samples.

### 2.4. Model Selection for a Given Classification Problem

For a variety of classification tasks, such as cancer subtyping based on gene expression data, we propose a method to evaluate the appropriateness of different models for the given problem. The following criteria are used to select the most suitable model: (1) *Classification accuracy*, which is quantified using the F1 score, calculated as:

(4) $$F_{1}=\frac{2\times Precision\times Recall}{Precision+Recall}$$

where precision and recall are defined as:

(5) $$Precision=\frac{TP}{TP+FP}\;and\;Recall=\frac{TP}{TP+FN}.$$

In these formulas, TP and FP represent the true positive and false positive counts, respectively. (2) *Classification stability* (CS), which assesses the model’s sensitivity to random mislabeling of N% of the samples. Specifically, it evaluates the difference in F1 score between the original and mis-labeled datasets. We calculate this as:

(6) CS=TP−TP’,

where TP’ is the same as TP but on the dataset with mislabeling. A small CS indicates that the model performs similarly on both original and altered data, implying robustness to noisy labels. (3) *Classification robustness* (CR), which evaluates the model’s ability to correctly classify mislabeled samples. This is calculated as:

(7) CR=TP’−(TP−k),

where k is the number of mislabeled samples that belong to the true positives. CR reflects the model’s capacity to recover from mislabeling and maintain accurate classification. A higher CR value signifies greater robustness to label noise.

To ensure reliable results, the process of randomly mislabeling 20% of the samples is repeated 100 times, and the average performance across all 100 modified datasets is used to derive the final metrics. In summary, the model selection process considers these factors: classification accuracy, stability, and robustness, each evaluated through specific performance metrics. Hence, for the selected model, we have

(8) $$\mathrm{CS}=\mathrm{TP}-\frac{\sum_{i}TP_{i}^{’}}{100},$$

(9) $$\mathrm{CR}=\frac{\sum_{i}(TP_{i}^{’}-(TP-x))}{100}=\frac{\sum_{i}TP_{i}^{’}}{100}-80\%\mathrm{TP}.$$

A possible approach to integrate the three objectives is by using a weighted sum of the three components:

(10) $$\alpha {F’}_{1}+\beta CS+\gamma CR$$

where α, β, γ are positive scaling factors, which will be determined empirically based on specific needs such as higher CS or higher CR. By default, α is set to 2 to amplify the weight of the F1’ score, ensuring that classifiers with better ${F’}_{1}$ performance are given more importance. β is set to −1, and γ is set to 0.1 to mitigate the impact of negative values.

### 2.5. Constructing an Integrative Pipeline

We have developed a two-level stacking pipeline [36] to incorporate the five classifiers, namely, LR, SVM, RF, LGBM, and XGB, via a meta-learner. The pipeline utilizes each of the classifiers to generate one prediction using the selected feature genes. The meta-learner then integrates the five predictions to produce the final classification, where the weights of the five classifiers are determined to optimize the classification result for each given problem, measured using *F*’1, CS, and CR (see Section 2.3). Specifically, the meta-learner is implemented using a gradient boosting algorithm [37], which effectively combines the weighted predictions by minimizing a loss function tailored to the specific classification metrics. The first-level weight calculation process is as follows:

(11) $${Score}_{i}=F{’1}_{i}+CS_{i}+CR_{i}$$

where $F’1_{i}$, $CS_{i}$, and $CR_{i}$ are scores by the *i*^th^ classifier. The weight of the *i*^th^ classifier, as determined by the second-level learner (meta-learner), is defined as follows:

(12) $$w_{i}=\frac{exp({Score}_{i})}{\sum_{j=1}^{5}exp({Score}_{j})}.$$

### 2.6. Comparative Evaluation of Predictive Performance, Interpretability, and Robustness of Models

To compare the quality of feature selection by our procedure and that by a state-of-the-art method, we have employed Recursive Feature Elimination (RFE) [38], a widely used method for feature selection.

We have compared our classification framework with both the machine learning and deep learning models using two classification problems (see Section 2.1), in the following categories: prediction accuracy, interpretability, model stability, and robustness. Here, for the deep learning models, we utilized a CNN-based architecture—LeNet [39]—and a deep neural network (DNN) model [40].

To evaluate the interpretability of a classification model, we compared it against two classes of widely used models: intrinsic interpretable white-box models [41] and post hoc surrogate models [42]. For intrinsic interpretability, focusing on the structural simplicity of a classification method, we selected two state-of-the-art white-box models: the Explainable Boosting Machine (EBM) [43] and RuleFit [44]. For post hoc interpretability, focusing on the ability to construct an interpretable approximation rather than to reconstruct the actual logical process for computing the results, we employed the game theory-based SHAP [45] technique. The evaluation criteria for interpretability included the contributions to classification results by feature genes and their functional annotation.

**Figure 1:**
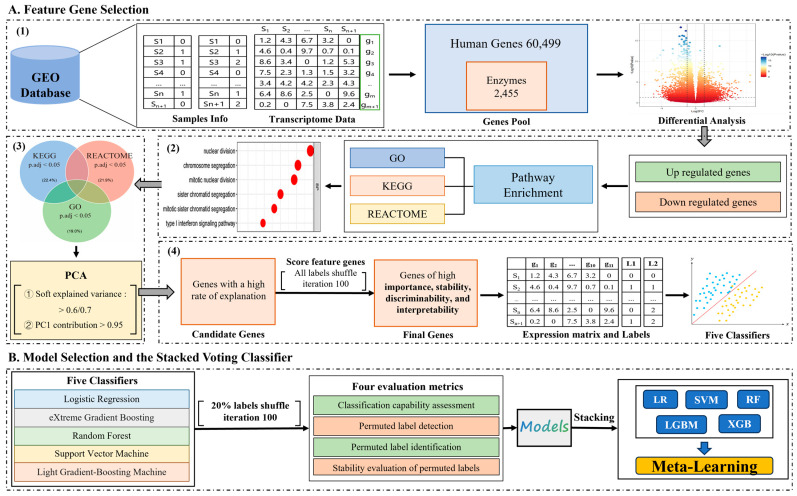
Construction of a feature selection and model selection framework. (**A**) This step includes four stages: (**1**) Identification of differentially expressed genes. (**2**) Identification of representative metabolic pathways in samples with distinct labels. (**3**) Identification of key genes within the representative metabolic pathways. (**4**) Identification of genes that are insensitive to adversarial samples. (**B**) Evaluation of model suitability for data based on accuracy, classification stability, and robustness. Subsequently, computation of the weights of primary classifiers based on scores derived from model selection, followed by the construction of a meta-classifier.

Figure 1 summarizes the procedure for feature and model selection. The test data, code, and the stacking pipeline are available for download at https://drive.google.com/drive/folders/17CWxRNx1Cdm17IIb_d8_lyg12VrRohTx (accessed on 20 January 2025).

## 3. Results

We assessed our classification framework using two classification problems, namely, the binary and ternary classification problems defined in Section 2.1, and compared its performance with the five state-of-the-art methods specified in Section 2.6.

### 3.1. Feature Gene Selection

To assess the importance scores of candidate feature genes, we evaluated their sensitivities to adversarial samples with each of the six classifiers, ours plus the five classifiers mentioned in Section 2.2. For each gene, we performed random permutations on the labels on a subset **S**’ of the whole sample set **S** 100 times and measured the changes in the gene’s importance scores [29]. A gene was considered *sensitive* to the adversarial samples if its importance scores in at least 95% of permutated sets were higher than its score in the un-permutated set, which was removed from further analyses.

**Figure 2:**
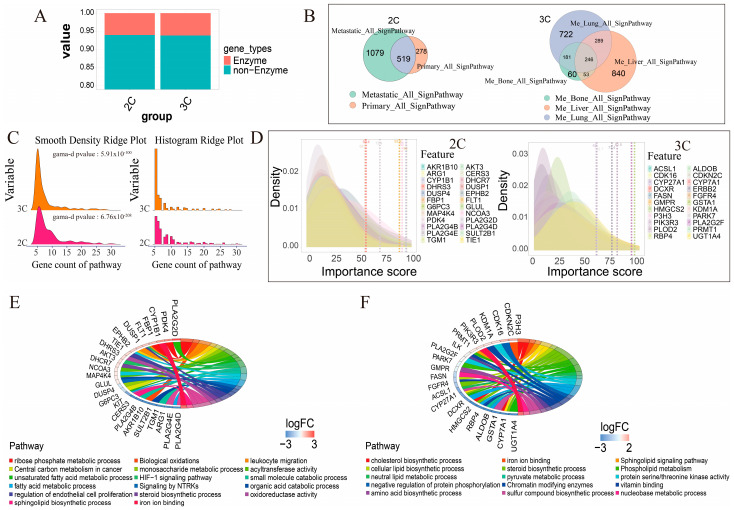
Feature evaluation. (**A**) Statistical overview of DEGs and enzyme genes in the binary and ternary datasets. (**B**) Occurrence statistics of the enriched pathways across sample groups with distinct labels in the binary and three-class datasets. (**C**) Gene counts in pathways meeting thresholding criteria in the binary and ternary datasets. Kolmogorov–Smirnov (KS) test for γ distribution is provided. (**D**) Distribution of importance scores over adversarial samples for the final selected features in the binary and ternary datasets. (**E**) Relationships between the final selected feature genes and cellular functions in the binary classification dataset. (**F**) Relationships between the final selected feature genes and cellular functions in the ternary classification dataset. Note: “2C” represents the binary classification dataset, and “3C” represents the ternary classification dataset.

Our past experience has shown that among all genes, both protein and RNA genes, enzyme genes tend to have the highest discerning power, which makes sense, as the behaviors of a disease are the direct acts of enzymes, rather than signaling or regulatory genes [46]. Hence, we used enzyme genes as the feature genes for classification problems. As the first step of feature selection based on *differentially expressed genes* (DEGs), we identified 215 enzyme genes among 3368 DEGs in the binary classification problem and 435 enzyme genes among 6657 DEGs in the tertiary dataset (Figure 2A). Furthermore, biological pathway enrichment analyses of the DEGs revealed that, for the binary classification problem, metastasized and primary cancers enriched 1079 and 278 functional pathways, respectively, and for the ternary dataset, pathway enrichment analyses showed that 294, 1182, and 1192 pathways are enriched by DEGs in cancer samples with metastases to bone, liver, and lung, vs. controls, respectively (Figure 2B).

For the classification problem, we selected among the enriched pathways that meet the selection criteria defined in Section 2.3. We noted that the number of genes per pathway across all the selected pathways follows a left-skewed γ distribution (α<1) (Figure 2C), based on which we defined the selection criteria equation $V’=0.7\times r^{\alpha },\;\alpha =0.5$. Here, V’ represents the filtering threshold for the first principal component (PC1).

For the selected feature genes based on the selected pathways (Section 2.4), we calculated each gene’s importance score over the adversarial samples. As shown in Figure 2D, genes meeting our criteria consistently exhibit lower importance scores over the adversarial samples than over the samples with the correct labels. Applying a majority-vote rule, we selected 25 enzyme genes for the binary classification problem and 23 for the ternary problem (Figure S1). Table S1 lists the ranking of the importance scores of the distinguishing enzyme genes for the two problems.

As illustrated in Figure 2E and Table S2, the relationships between the feature genes and biological pathways reveal distinct differences between metastatic and non-metastatic cancers. Notably, genes such as AKT3 [47], involved in the hypoxia-stress-induced HIF-1 signaling pathway, CYP1B1 [48], which plays a role in oxidative stress and iron accumulation, and PLA2G2D [49], which is implicated in fatty acid synthesis, were key discriminants. These findings align with evidence showing that primary cancer sites are often characterized by hypoxic and highly oxidative environments [50,51,52,53]. Specifically, iron accumulation is associated with intracellular alkalosis, while fatty acids act to mitigate this alkalosis [54,55]. Furthermore, 80% (19/25) of the selected enzymes produce H^+^ in the reactions they catalyze, which is consistent with the upregulation of acidifying metabolic reprogramming in cancer [54,55].

In the ternary classification task, biological pathways differentiating the three metastasis sites—liver, lung, and bone—highlighted processes such as cholesterol synthesis, lipid metabolism, oxidoreductase activity, and pyruvate metabolism (see Table S2, Figure 2F). These findings are consistent with known characteristics of the three organs. For example, immune response-related pathways (ILK [56]), high-energy phosphate transfer reactions (PLA2G2F in liver [57]), oxidation–reduction processes (GSTA1 in lung [58]), the regulation of nerve cell differentiation (RBP4 in lung), and erythrocyte differentiation and melanosome organization in bone metastases (PIK3R3 [59]) were particularly prominent. Additionally, cholesterol accumulation, seen in the involvement of CYP7A1 [60], was common across all metastatic sites [61,62].

**Figure 3:**
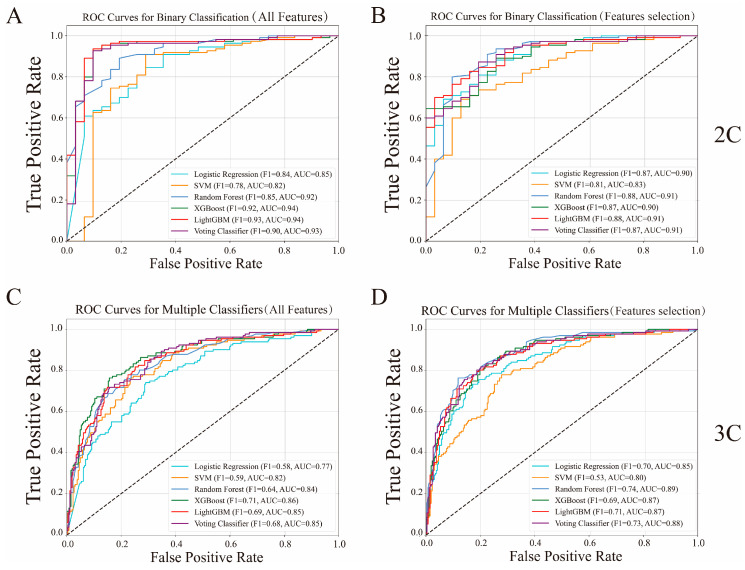
Comparison of prediction performance for five classifiers and the stacking voting classifier using all genes vs. selected feature genes in two problems. (**A**) Prediction performance in the binary classification using the full gene set (60,499 genes). (**B**) Prediction performance in the binary classification using our 25 enzyme genes. (**C**) Prediction performance in the ternary problem using the full gene set (60,499 genes). (**D**) Prediction performance in the ternary problem using our 23 enzyme genes. Note: “2C” represents the binary classification dataset, and “3C” represents the ternary classification dataset.

To further validate the effectiveness of our feature selection approach, we compared the performance of the five models trained using the selected enzyme genes against models trained on the full feature set (60,499 genes). In the binary classification problem, as shown in Figure 3A,B, LR, SVM, and RF achieved 3–4% higher F1 scores with our selected features, while LGBM and XGB exhibited a 5% decrease. Similarly, in the ternary problem (Figure 3C,D), all models except SVM showed performance improvements of 2–12% with our selected features.

### 3.2. Model Assessment

**Figure 4:**
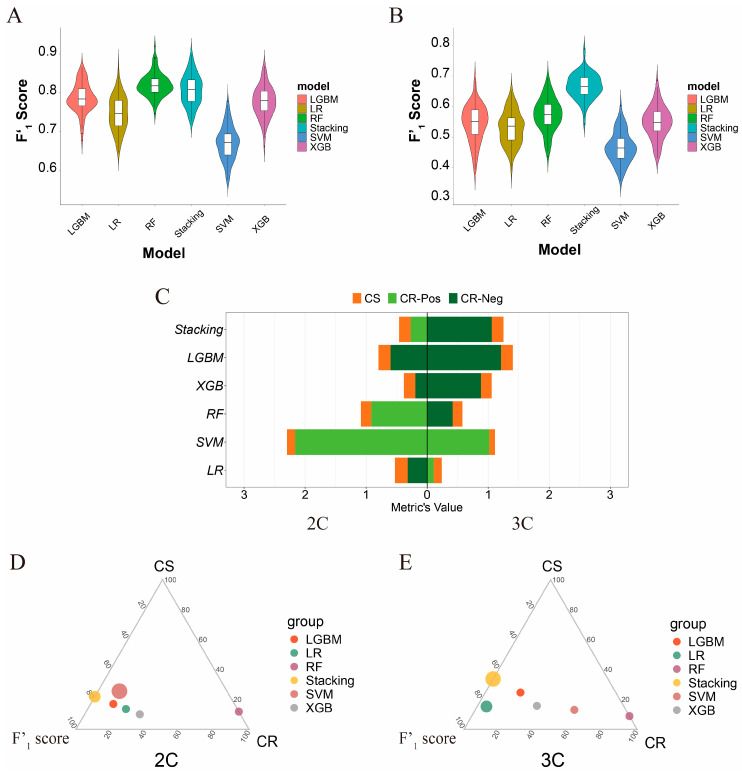
Model selection. (**A**) ${F’}_{1}$ score evaluation of six models in the binary problem. The square box represents the variance of the ${F’}_{1}$ score over 100 iterations. (**B**) ${F’}_{1}$ score evaluation of six models in the ternary problem. (**C**) Classification stability and robustness of the six models. (**D**) Comprehensive evaluation of ${F’}_{1}$ score, CS, and CR metrics of the six models in the binary problem. (**E**) Comprehensive evaluation of ${F’}_{1}$ score, CS, and CR metrics of the six models in the ternary problem.

We then used the procedure given in Section 2.4 for model selection from the five candidate classifiers. Figure 4 shows the performance in terms of performance accuracy, stability, and robustness in the two classification problems of the five classifiers, which are detailed in Table 1. Overall, in the binary problem, RF and the stacking voting classifier exhibit the best predictive performance, while in the ternary problem, the stacking voting classifier demonstrates the highest predictive performance (Figure 4A,B).

Figure 4C and Table 1 present the classification stability of the five classifiers in the two problems. In both the binary and ternary problems, SVM has the best performance. In terms of classification robustness, SVM also exhibits the highest performance in both the binary and multiclass datasets.

Overall, when the F1′ scores for the five classifiers on the unmodified label samples are high, SVM is the preferred model. However, when the ${F’}_{1}$ scores are suboptimal, the stacking voting classifier emerges as the superior choice (Figure 4D,E).

### 3.3. Performance Analysis of a Stacking-Based Voting Meta-Classifier

**Figure 5:**
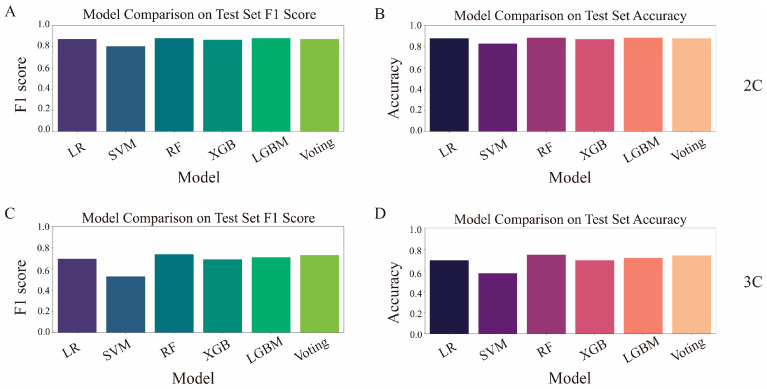
F1-score and accuracy statistics of the stacking voting classifier in the test set by our framework. (**A**) F1-score statistics of our classifier in the binary problem. (**B**) Accuracy statistics of our classifier in the binary problem. (**C**) F1-score statistics of our classifier in the ternary problem. (**D**) Accuracy statistics of our classifier in the ternary problem.

Due to imbalance issues across sample groups with different labels in both problems (the binary problem: 368:102; the ternary problem: 207:77:52), the F1 score, which is more suitable for imbalanced data, is reported for the test set (without adversarial examples). In both the binary and ternary problems, our classifier ranks in the top two performers, (Figure 5A,B). The prediction accuracy in the test set shows a similar trend to the F1 score (Figure 5C,D).

### 3.4. Comparison Between General-Purpose Feature Selection and Our Feature Selection

We compared our feature selection procedure with a widely used general-purpose feature selection procedure, the REF approach, aiming to gain insights about the level of improvement that domain-specific knowledge can provide for general-purpose feature selection.

**Figure 6:**
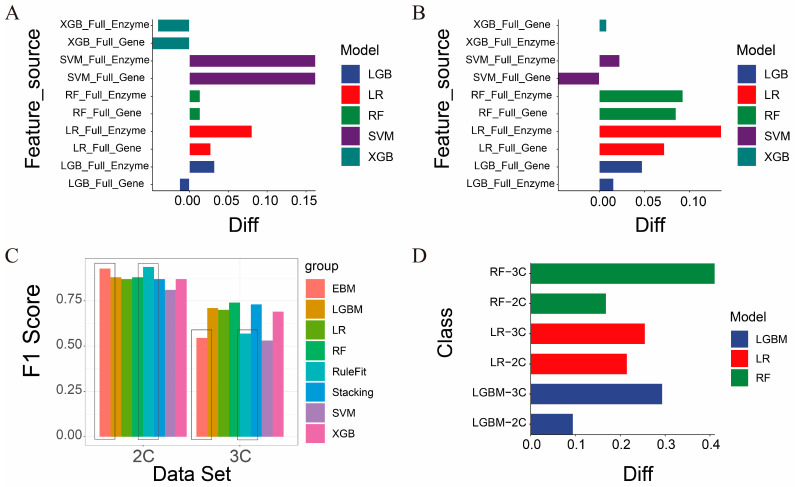
Comparison of prediction performance between our model, RFE, interpretable white-box models, and SHAP. (**A**) Difference in F1 scores between features selected by our framework and the top features selected by RFE in the binary classification. Positive values indicate higher F1 scores achieved by our framework. (**B**) Difference in F1 scores between features selected by our framework and the top features selected by RFE in the ternary classification. Positive values indicate higher F1 scores achieved by our framework. (**C**) F1-score comparison between features selected by our framework and the top features selected by white-box models (EBM and RuleFit) in both binary and ternary classification problems. (**D**) Difference in F1 scores between features selected by our framework and the top features selected by SHAP in the binary and ternary classification problems. Positive values indicate higher F1 scores achieved by our framework.

We compared the classification results obtained using RFE with those using our feature selection method. As shown in Figure 6A,B and Table S3, across all five models, our feature selection method outperforms RFE in terms of predictive performance, except for XGB in the binary classification. In the ternary problem, our feature selection method also outperforms RFE in terms of predictive performance, except for SVM.

Similarly, among the 25 selected feature genes for the binary problem and the 23 selected genes for the ternary problem, we observe that the majority were regulatory non-enzyme protein-coding genes and non-protein-coding genes, irrespective of the classifier used (Table S4). Furthermore, within these genes, only the top genes selected by LGBM’s RFE demonstrated excellent resistance to adversarial perturbations in other models, whereas the genes selected by the other models exhibited poor resistance to adversarial samples (Table S5).

### 3.5. Comparison of Model Interpretability

#### 3.5.1. Comparison with the White-Box Models

White-box models derive their interpretability from both the predictive performance of the model and the ranking of feature importance; generally, better prediction performance and higher-ranked feature importance correspond to more understandable feature explanations [63]. Accordingly, we used EBM and RuleFit to select the same number of feature genes as our framework from the 60,499 genes in the dataset: 25 genes for the binary classification dataset and 23 genes for the ternary classification dataset. As shown in Figure 6C, for the simpler binary classification task, EBM and RuleFit exhibit the best performance (Table S6), whereas they clearly underperform in the more complex ternary classification task compared to the stacking voting classifier. Furthermore, when adversarial samples are introduced, the F1′ score of the stacking voting classifier significantly outperforms both EBM and RuleFit in both binary and ternary settings. After considering both CS and CR, the stacking voting classifier remains the top performer (Table 2).

Regarding feature gene interpretability, EBM provides more reasonable selections than RuleFit, as approximately 30% of the genes identified by RuleFit lack functional annotations (Table S7). In contrast, while both EBM and RuleFit predominantly select non-enzyme genes (95%), these genes, such as SMIM2 antisense RNA 1, are not notably linked to tumor hallmarks, unlike the feature genes identified by our framework (Table S7).

#### 3.5.2. Comparison with SHAP Models

Due to the substantial memory and time requirements for training SHAP with SVM and XGB models—exceeding 1 TB of memory—we limited our SHAP analysis to LR, LGBM, and RF results. The interpretability of SHAP models similarly relies on feature importance [64]. Consistent with the RFE results, when training models using an equivalent number of our selected features, we found that for both binary and ternary classification problems, the F1 scores based on our feature selection surpassed those obtained using SHAP by at least 10% (Figure 6D, Table S8). Similarly, to analyze the feature interpretability provided by SHAP, we examined the top features it ranked as highly important. We observed that 60% of these features were non-coding protein genes or pseudogenes (Tables S9 and S10).

#### 3.5.3. Comparison with Neural Network Models

To assess the performance of neural network models within our framework, we first evaluated the LeNet and DNN models using a complete gene set comprising 60,499 genes. As shown in Table S8, the prediction accuracies of LeNet and DNN for the binary classification task were 0.7882 and 0.815, respectively. For the multiclass classification task, the accuracies of LeNet and DNN were 0.29881 and 0.5291, respectively (Table S11).

Further evaluation of the impact of adversarial samples on the neural network models revealed that, as shown in Table 2, the DNN model outperformed all other models in the binary classification dataset. However, in the multiclass dataset, the performance of both neural networks significantly declined, with F1 scores of 0.2344 and 0.2653, respectively.

**Figure 7:**
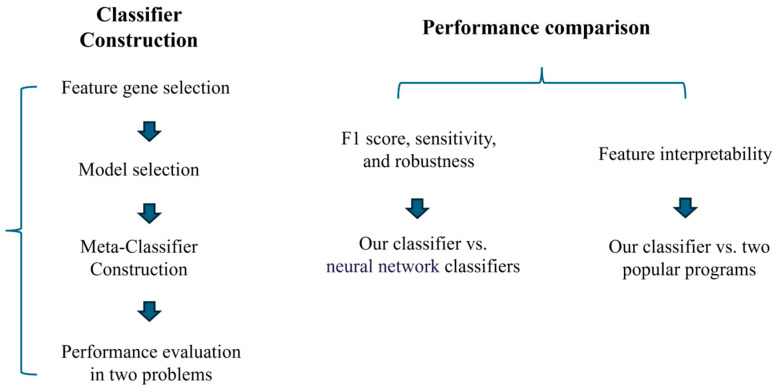
Overview of our algorithmic framework.

Additionally, we have provided a summary flowchart of the analysis framework and evaluation process in Figure 7.

## 4. Discussion

This work introduces a machine learning framework designed specifically for classification tasks using transcriptomic data. The goal is to assist researchers in effectively combining biological insights with machine learning models that are best suited to the specific nature of the classification problem. The biological knowledge is incorporated through pathway-based analyses [65]. For selecting the optimal model, two essential pieces of information are needed: the most relevant features and models that exhibit the necessary characteristics for superior performance. This method not only achieves high prediction accuracy but also highlights features with significant interpretability. Moreover, by employing five base learners within a stacked ensemble framework, guided by comprehensive model selection criteria, we developed a meta-model that guarantees robust performance, even in the presence of adversarial perturbations.

Additionally, we compared our framework with current popular interpretable white-box models, such as EBM [43] and RuleFit [66], as well as post hoc interpretability proxy models like SHAP [45]. Our framework demonstrated superior interpretability by selecting biologically meaningful and robust features that are directly linked to relevant metabolic pathways [67]. We have demonstrated that our overall classification framework achieved higher prediction accuracies compared to state-of-the-art methods, highlighting the effectiveness of our integrated approach in enhancing both the explainability and performance of classification models in transcriptomic data analysis [68].

Furthermore, we explored the performance of neural network models within our framework. Neural networks exhibited notable advantages in terms of robustness to adversarial samples, as evidenced by their high CR values in both the binary and ternary classification tasks. This robustness suggests that neural networks can maintain performance integrity even when faced with perturbed or noisy data [69]. However, their prediction accuracy in the ternary classification problem was significantly hampered, primarily due to the limited size of the training dataset. The scarcity of samples restricts the neural networks’ ability to generalize effectively across multiple classes, resulting in suboptimal performance compared to more traditional machine learning models [70]. This trade-off between robustness and predictive accuracy underscores the challenges of applying deep learning techniques to small-sample omics data, emphasizing the need for larger datasets or enhanced training strategies to fully leverage the strengths of neural networks in such contexts [71].

It is important to mention that our current study is somewhat exploratory in nature. As we continue to address a broader range of biological classification problems, we plan to expand our analyses systematically by incorporating additional models, potentially grouped into categories such as tree-based and SVM-like techniques, among others. This will allow us to deepen our understanding of which types of classification problems are best suited to specific classes of methods [72]. Moreover, future work will consider a broader set of performance metrics, including the effects of noise and missing data on classification tasks, as well as identifying the most effective techniques for addressing these challenges [73].

In summary, our research introduces a comprehensive and flexible framework for feature and model selection, enabling the identification of the most suitable models based on the specific traits of the data. This work contributes to improving classification accuracy, stability, and robustness, particularly in bioinformatics and other fields that deal with large, complex datasets. Future developments will refine and extend this framework, making it applicable to a wider variety of datasets and classification tasks. Our methodology provides a practical approach for data analysts, especially those who may not be deeply familiar with the mathematical foundations of machine learning algorithms, allowing them to select the optimal model for their challenges and maximize the potential of machine learning in data-heavy environments.

## 5. Conclusions

We have designed a framework for feature and model selection tailored to omics data classification tasks, alongside a meta-classifier learning approach that ensures both high classification performance and the interpretability of the results. This framework is implemented through a multi-step selection process, involving gene selection, model identification, and the aggregation of meta-classifiers. The interpretability is achieved by focusing on pathways that are well-characterized, have strong discriminative power, and play a key role in explaining the primary variance of gene expression across a wide range of samples.

We have shown the efficacy of this framework in addressing two distinct classification challenges: one involving binary classification and the other a three-class classification task. We believe this framework has the potential to provide a versatile solution for both feature and model selection in a wide range of omics classification applications.

## Figures and Tables

**Table 1:** Performance measures for model selection, in terms of performance accuracy, classification stability, and robustness.

Model	Labels	${F’}_{1}$ Score	CS	CR	Final Score
LR	2C	0.7492	0.2073	−0.3200	1.2591
SVM	2C	0.6739	0.1327	2.1650	1.4316
RF	2C	0.8221	0.1710	0.9150	1.5647
XGB	2C	0.7892	0.1869	−0.1950	1.3720
LGBM	2C	0.7791	0.1969	−0.6000	1.3013
Stacking	2C	0.8097	0.1904	0.2700	1.4560
LR	3C	0.5262	0.1325	0.1067	0.9306
SVM	3C	0.4641	0.0948	1.0167	0.9351
RF	3C	0.5692	0.1617	−0.4167	0.9350
XGB	3C	0.5452	0.1786	−0.8800	0.8238
LGBM	3C	0.5412	0.1928	−1.2100	0.7686
Stacking	3C	0.6659	0.1902	−1.0600	1.0356

**Table 2:** Comparison of the stacking voting classifier, interpretable white-box models, SHAP, and neural network models in terms of performance accuracy, classification stability, and robustness.

Method	Labels	F1_Score	CS	CR	Final Score
Stacking	2C	0.8097	0.1904	0.2700	1.4560
EBM	2C	0.6869	0.18017	0.8699	1.2806
RuleFit	2C	0.6414	0.2215	−2.555	0.8058
LeNet	2C	0.6286	0.1145	2.633	1.4060
DNN	2C	0.6684	−0.0018	2.246	1.5632
Stacking	3C	0.6659	0.1902	−1.0600	1.0356
EBM	3C	0.5366	0.16255	−0.32	0.8787
RuleFit	3C	0.5470	0.16258	−0.4633	0.8671
LeNet	3C	0.2344	0.00061	1.9013	0.6583
DNN	3C	0.2653	−0.0006	1.914	0.7226

## Data Availability

The data supporting the reported results can be found at https://drive.google.com/drive/folders/1ik6-qABgVwk_lnoXubN9ltpXYVfLDrGG (accessed on 19 January 2025).

## Supplementary Materials

The following supporting information can be downloaded at: https://www.mdpi.com/article/10.3390/genes16030297/s1, Figure S1 and Tables S1–S11.
