# Supplementary material for: Optimizing Model Performance and Interpretability: Application to Biological Data Classification
Source: Genes (Basel). 2025 Feb 28;16(3):297. doi: 10.3390/genes16030297 (PMC11942234; doi:10.3390/genes16030297)
Supplement: Supplementary file 1 [file genes-16-00297-s001.zip › Figure S1.pdf]

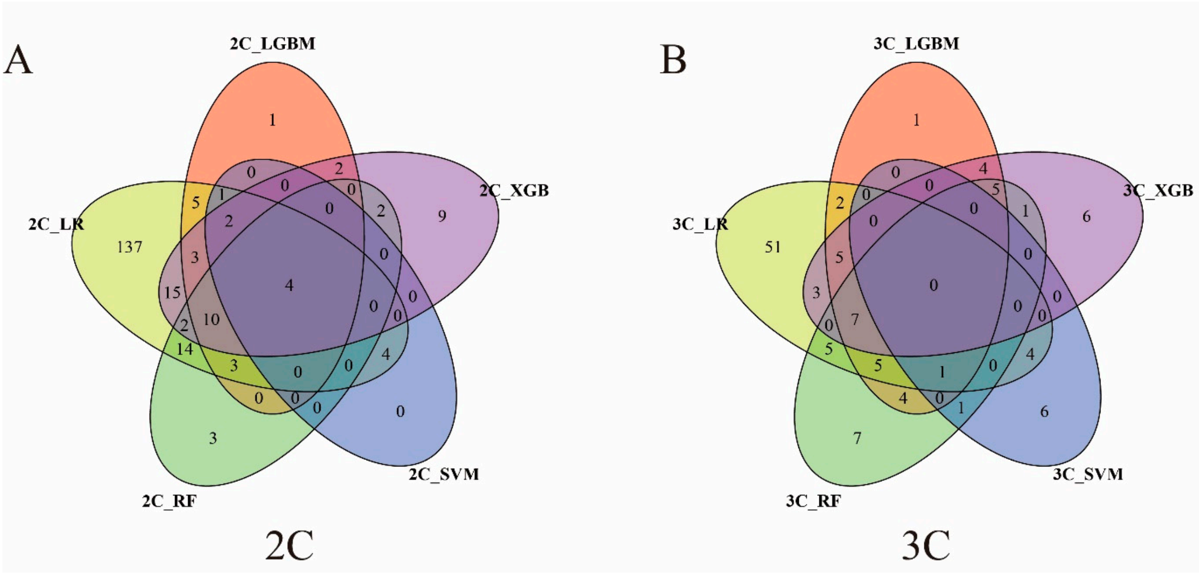

Figure S1. Venn diagram statistics of genes filtered by null importance across five classifiers. A. Venn diagram statistics of genes filtered by null importance across five classifiers in the binary classification dataset. B. Venn diagram statistics of genes filtered by null importance across five classifiers in the ternary classification dataset.
